# Troxerutin attenuates LPS-induced inflammation in BV2 microglial cells involving Nrf2 activation and NF-κB pathway inhibition

**DOI:** 10.22038/ijbms.2025.87692.18941

**Published:** 2025

**Authors:** Shengnan Ma, Hongguang Fan, Jianhong Zhang, Lijun Wang, Haiwen Shi, Changjun Li

**Affiliations:** 1 Department of Pharmacy, Tianjin Fourth Central Hospital, Tianjin 300140, China; 2 Department of Neurology, Tianjin Fourth Central Hospital, Tianjin 300140, China

**Keywords:** Anti-Inflammatory agents, Lipopolysaccharides, Microglia, Neuroinflammation, NF-kappa B, Nrf2 transcription factor

## Abstract

**Objective(s):**

Microglial cell-mediated neuroinflammation is a key driver of central nervous system (CNS) homeostasis and a significant risk factor for neurodegeneration and development of neurological diseases. We assessed whether troxerutin (TX) exerts anti-neuroinflammatory effects in lipopolysaccharide (LPS)-stimulated BV2 microglia and explored its mechanism.

**Materials and Methods:**

To investigate the suppressive action of TX on M1 polarization, BV2 cells were stimulated with LPS and then treated with TX or minocycline (MINO). Cell viability was assessed via Cell Counting Kit-8 (CCK-8), and inflammatory cytokines were measured by quantitative polymerase chain reaction (qPCR) and enzyme-linked immunosorbent assay (ELISA). Furthermore, the nuclear factor erythroid 2-related factor 2 (Nrf2)/nuclear factor-kappa B (NF-κB) signaling pathway was analyzed by Western blotting (WB) to elucidate the molecular mechanism of the anti-neuroinflammatory activity of TX.

**Results:**

TX inhibited the expression of interleukin-6 (IL-6) and interleukin-1β (IL-1β), as well as the secretion of IL-6 and tumor necrosis factor-α (TNF-α). Additionally, TX accelerated the release of transforming growth factor-β (TGF-β) and cluster of differentiation 206 (CD206) in BV2 microglia exposed to LPS. TX regulated the neuroinflammatory response by blocking phosphorylation of NF-κB and inhibitor of kappa B alpha (IκBα) mediated by LPS stimulation and inducing Nrf2 and heme oxygenase-1 (HO-1) protein expression.

**Conclusion:**

TX suppresses pro-inflammatory induction after LPS stimulation of BV2 microglia, which may be related to the NF-κB inhibition and accelerated HO-1/Nrf2 activation. These findings pinpoint the potential therapeutic potential of TX in inflammation-induced neurodegenerative diseases.

## Introduction

TX, popularly known as vitamin P4, is a bioflavonoid rutin derivative abundantly expressed in flower buds of *Sophora japonica*, tea, coffee, cereal grains, and several different vegetables and fruits (1). TX exhibits favorable biological properties (2), including but not limited to anti-oxidant (3), anti-inflammatory (4), anti-hyperglycemic (5), antiapoptotic (6), anticancerous (7), antithrombotic, and vasoprotective activities (8). Studies have confirmed its neuroprotective effects against neurological disorders, as it exerted antidepressant functions (9), alleviated diabetes-associated cognitive decline (10), and improved spatial cognition and memory in a rat model of Alzheimer’s disease (AD) (11–13).

Accumulating evidence emphasizes a strong link between uncontrolled chronic neuroinflammation and the progression of neurodegeneration, as observed in AD (14), Huntington’s disease (15), Parkinson’s disease (PD) (16), and multiple sclerosis (15). Microglial cell-mediated neuroinflammation is a key driver of homeostasis in the CNS and a known risk factor for neurodegeneration (17, 18). Upon activation, microglia/macrophages get phenotypically polarized from their resting M0 state into either M1 or M2 phenotype (19). M1 cells are associated with the functions of pro-inflammatory molecules, as they release tumor necrosis factor-alpha (TNF-α), IL-1β, and IL-6 (20, 21) and orchestrate inflammation and cytotoxic responses (22, 23). On the contrary, M2 microglia/macrophages mediate an anti-inflammatory response and generate anti-inflammatory molecules, some of which are interleukin-10 (IL-10), interleukin-4 (IL-4), and TGF-β (24), which contribute to tissue repair and remodeling (25). Thus, a potential prevention and treatment strategy for neurodegenerative disease management involves the suppression of microglial overactivation through blockade of endogenous inflammatory factor production (26).

Currently available drugs to combat degenerative brain diseases pose the risk of toxic side-effects. Hence, researchers are driven to find measures for inflammatory response management through the use of relatively safer and naturally available products. Studies thus far on natural products such as phenylpropanoids, flavonoids, terpenoids, alkaloids, and their derivatives have confirmed their abilities to alleviate pathological changes observed in AD. These molecules were shown to impart multiple functions such as inhibition of acetylcholinesterase, reduction of tau and amyloid aggregation, amelioration of neuroinflammation, alleviation of amyloid-beta peptide 1-42 (Aβ1-42)-induced neurotoxicity, and inhibition of β-site amyloid precursor protein cleaving enzyme 1 (BACE-1) (27).

To this end, this study aimed to investigate whether TX exerts anti-neuroinflammatory effects in LPS-stimulated BV2 microglia, with a specific focus on elucidating its underlying mechanisms via modulation of the Nrf2/HO-1 and NF-κB signaling pathways. By delineating the regulatory effects of TX on microglial polarization and cytokine secretion, this work seeks to provide novel insights into its potential as a therapeutic agent for neuroinflammatory and neurodegenerative disorders.

## Materials and Methods

### Reagents

LPS (Batch no. 210I034) and radioimmunoprecipitation assay (RIPA) buffer (high) (Batch no. 20221112) were purchased from Solarbio (Beijing, China). Dulbecco’s Modified Eagle Medium (DMEM) (Batch no. FG2212) and Fetal Bovine Serum (FBS) (Batch no. YS210414) were obtained from EallBio Life Sciences (Beijing, China). Mouse IL-6 ELISA Kit (Batch no. 20220942A) was purchased from Mlbio Company (Shanghai, China). Mouse TNF-α ELISA Kit (Batch no. 220532M) was obtained from Meimian Industrial Co., Ltd (Jiangsu, China). Nuclear and Cytoplasmic Protein Extraction Kit (Batch no. 110922230706) was purchased from Beyotime Biotechnology (Shanghai, China). Enhanced chemiluminescence (ECL) (Batch no. ATVJ28071) reagent was from Abbkine Scientific Co., Ltd (Wuhan, China). In addition, IκBα (Batch no. 11), phospho-IκBα(Ser32) (Batch no. 18), and NF-κB p65 (Batch no. 9) antibodies were purchased from Cell Signaling Technology, Inc. (Danvers, MA, United States), phospho-NF-κB p65/RelA-S536 (Batch no. 6100000384), glyceraldehyde-3-phosphate dehydrogenase (GAPDH) (Batch no. 3507443007) antibodies, and horseradish peroxidase-conjugated (HRP-conjugated) goat anti-rabbit secondary antibodies (Batch no. 9300014001) were procured from ABclonal Technology Co. Ltd. (Wuhan, China). Lamin B1 (Batch no. 20230810), HO-1/HMOX1 (Batch no. 20230810), Kelch-like ECH-associated protein 1 (KEAP1) (Batch no. 20230810) , and NRF2 (Batch no. 20230810) antibodies were obtained from Bioswamp (Wuhan, China).

### Cultures

BV2 cells supplied by Cell Resource Center, EallBio Life Sciences, were authenticated using short tandem repeat (STR) profiling within the last 3 years. The cells were confirmed, before experimentation, to be free from mycoplasma. For cell culture, BV2 microglia were cultured in DMEM supplemented with 10% FBS and antibiotics (100 U/ml penicillin and 100 µg/ml streptomycin). Cell cultures were maintained in a humidified atmosphere under normal growth conditions (5% CO_2_ and 37 °C).

In the present study, BV2 cells were categorized into the following five groups: i) Control group, Bv2 cells were cultured in normal medium; ii) LPS group, Bv2 cells were cultured in medium containing LPS; iii) TX (10 µM) + LPS group, BV2 cells were incubated with TX (10 µM) for one hour followed by co-treatment with LPS; iv) TX (50 µM) + LPS group, BV2 cells were incubated with TX (50 µM) for one hour followed by co-treatment with LPS; v) MINO + LPS group, BV2 cells were incubated with MINO (10 µM) for one hour and then subjected to LPS.

### Viability assay

Cellular viability following test experiments was determined using the CCK-8 (Lot No. F2211, EallBio Life Sciences, Beijing, China) assay. The CCK-8 assay was selected for its higher sensitivity, water-soluble formazan product (avoiding crystal dissolution steps), and compatibility with kinetic studies compared to traditional MTT (28). In this assay, WST-8 is reduced by cellular dehydrogenases to an orange formazan dye, with absorbance (450 nm) directly proportional to viable cell count. In brief, cells were seeded in 96-well plates at 5 × 10^3 ^cells/well, allowed to attach, and then exposed to TX (0.1, 1, 10, 100, 500, and 1000 μM) dissolved in DMEM. After 24 hr, each well was incubated with CCK-8 reagent (1:100) for one hour, and the absorbance at 450 nm wavelength was measured.

### ELISA

BV2 cells (2 × 10^5^) were seeded into 12-well plates and incubated at normal growth conditions (37 °C for 24 hr). For the test experiment, the cells were exposed to TX and LPS (prepared in DMEM), as appropriate. After treatment for 24 hr, 1 ml of cell supernatant was centrifuged (2000 ×*g* for 20 min) and employed to determine IL-6 and TNF-α concentrations using an enzyme immunoassay as indicated (23). 

### RT-qPCR

BV2 cells were subjected to RNA isolation (Ultra-pure total RNA extraction kit, Batch no. 20211153), cDNA synthesis (cDNA first-strand synthesis kit, Batch no. 20220707), and real-time qPCR (2× SYBR Green PCR Mix, Batch no. 20220104) following manufacturers’ instructions (Hangzhou Simgen Biotechnology Co., Ltd., China). The Primer Express Software was used to design primer sequences listed in Table 1. Roche LightCycler 96 SW software was employed to determine the cycle threshold for all reactions. qPCR was performed in triplicate, with each experiment repeated at least twice. Normalization of target gene expression was conducted relative to housekeeping β-actin gene expression using the 2^−ΔΔCt ^method. The control value was set to 1 before calculation of the values displayed in Figure 3(C-F). A melting curve analysis was conducted to determine all PCR product specificity (25).

### Protein extraction and western blotting

Each test sample was lysed in a lysis buffer, and the protein concentration in the sample was analyzed by bicinchoninic acid (BCA) protein assay. Equal protein amounts from all samples were subjected to sodium dodecyl sulfate polyacrylamide gel electrophoresis (SDS-PAGE) separation. The obtained separation of protein bands was transferred onto Polyvinylidene fluoride (PVDF) membranes, which were blocked with 5% non-fat milk. After blocking non-specific binding, the membranes were probed with appropriate primary antibodies (All primary antibodies were diluted 1:1,000). Following overnight incubation at 4 °C, they were rinsed and incubated for one hour at room temperature with appropriate HRP-conjugated secondary antibodies (1:10,000) (29). The reaction was visualized by chemiluminescence (ChemiDoc^TM^ XRS System, version 721BR08034, Bio-Rad, Hercules, California, United States).

### Statistical analysis

Results are expressed as the means ± Standard Deviation (SD) of triplicates. Statistical significance was tested by one-way analysis of variance (ANOVA) followed by Tukey’s multiple comparison test using GraphPad Prism 5 (San Diego, United States). *P*<0.05 was considered significant difference.

## Results

### TX does not alter BV2 cell viability

We performed a CCK-8 assay to determine if TX affects BV2 cell viability. BV2 cells were incubated in the presence of varying concentrations (0.1, 1, 10, 100, 500, and 1000 µM) of TX, and the concentration below 100 µM failed to alter the cell growth (Figure 2A). In addition, we examined BV2 cell viability after their exposure to 1 µg/ml LPS (Figure 2B). Upon stimulation with LPS for 24 hr, the synapses of BV2 cells became longer, and the cytosol became larger and showed a typical amoeba-like appearance (30). However, TX treatment prevented these morphological alterations (Figure 2C). MINO was used as the positive control (31).

### TX reduces LPS-stimulated pro-inflammatory molecule generation in BV2 cells

We investigated whether TX affects IL-6 and TNF-α generation following stimulation of BV2 microglia with LPS and determined its anti-neuroinflammatory potential. The ELISA results shown in Figure 3 (A and B) confirmed the significant elevation in IL-6 and TNF-α in the group treated with LPS as compared with the control. However, TX treatment reduced the accelerated IL-6 and TNF-α generation upon LPS stimulation (*P*<0.01), similar to MINO.

### TX modulates the expression of cytokines in LPS-activated BV2 cells

We performed RT-qPCR to investigate the expression of pro-inflammatory molecules (IL-6 and IL1-β), chemokines (CD206), and neurotrophic factors (TGF-β). In comparison with the control group, the LPS-treated group showed significant up-regulation in IL-6 and IL1-β mRNAs. However, this effect on cytokine production was significantly reversed after TX treatment (*P*<0.01; Figure 3C, D). Further, LPS-treated cells showed a significant down-regulation in TGF-β and CD206 mRNAs relative to control cells, while TX treatment abrogated this effect and significantly up-regulated their levels (*P*<0.05; Figure 3E, F). And the increase in CD206 mRNA expression level was more pronounced compared to MINO. These observations demonstrate the TX-mediated IL-6 and IL-1β inhibition and TGF-β and CD206 up-regulation. 

### TX abrogates the increase in NF-κB p-p65/p65 and p-IκBα/IκBα after LPS stimulation of BV2 cells

NF-κB regulates the expression of various genes that participate in immune and inflammatory reactions (32). The LPS-induced microglial cell activation results in IκBα degradation and phosphorylation and the subsequent nuclear translocation of free NF-κB, where it triggers activities of inflammatory mediators such as IL-6 and Cyclooxygenase-2 (COX-2) (33). As shown in Figure 4, in the group treated with LPS, the ratio of p-IκBα/IκBα and NF-κB p-p65/p65 increased, but this increase was significantly abrogated in the TX treatment group (*P*<0.05). MINO also inhibited NF-κB phosphorylation, but there was no obvious effect on IκBα phosphorylation. These findings are suggestive; the beneficial effects of TX on the management of inflammation may be involved with the inhibition of NF-κB signaling. 

### TX regulates KEAP1-NRF2/HO-1 signaling in BV2 cells

Oxidative stress is essential in the pathological process of reperfusion of injured brain tissue (34). Considering KEAP1-Nrf2/HO-1 as a classical anti-oxidative pathway, we sought to investigate the impact of TX on the expression of Nrf2, KEAP1, and HO-1 in LPS-activated BV2 microglia. The TX group showed significantly higher nuclear-Nrf2 and HO-1 levels and lower cyto-Nrf2 and KEAP1 levels than the LPS-activated group (*P*<0.05; Figure 5), which was perhaps different from the mechanism of MINO on the Nrf2 signaling pathway. Thus, TX attenuates LPS-induced oxidative damage in microglia, which may be related to modulation of KEAP1-NRF2/HO-1 signaling. 

## Discussion

Herein, we demonstrate that TX inhibits the production of neuroinflammatory factors, including IL-6 and TNF-α, in BV2 microglia following LPS insult. Further, we show that TX, under the same conditions, down-regulates IL-6 and IL-1β expression while simultaneously escalating TGF-β and CD206 release. These findings are indicative of the suppressive action of TX on M1 polarization, which is mediated by inhibition of TNF-α, IL-6, and IL-1β, the known markers of M1 activation. Consequences of these events include potent inhibition of neuroinflammation. We also delineate the molecular mechanism of the anti-neuroinflammatory activity of TX by investigating its effects on NF-κB, IκBα, and KEAP1-NRF2/HO-1 signaling, which may be different from MINO on the anti-neuroinflammatory mechanism. 

As a transcription factor, NF-κB participates in Toll-like receptor 4 signaling and controls the expression of several factors associated with immune reaction and inflammation (32). NF-κB, under normal physiological conditions, forms a complex with IκB and is restricted to the cytoplasm but gets phosphorylated and subsequently ubiquitinated at IκB in response to various inflammation-related stimuli (e.g., IL-1β, TNF-α, or LPS). This phenomenon results in its activation (35). Upon LPS stimulation of microglia, the phosphorylated NF-kB dimers dissociate from IκB and translocate into the nucleus, wherein they promote the release of proinflammatory cytokines and consequently contribute to the vicious cycle of neuroinflammation (36). We determined whether the modulatory effects of TX on inflammatory mediator expression are associated with NF-κB expression alteration. We found that TX abrogated IκB and p65 phosphorylation in BV2 cells exposed to LPS. This action eventually repressed the p65 nuclear translocation process. Similar to TX, 4-methoxycinnamyl p-coumarate has been shown to reduce neuroinflammation by blocking NF-κB and MAPK pathways, while amentoflavone inhibits TLR4/MyD88/NF-κB signaling, highlighting the conserved role of NF-κB suppression in anti-neuroinflammatory mechanisms (37, 38).

Nrf2 belongs to the Cap’n’collar (CNC) family (39) and channelizes the regulation of various cellular phenomena, including redox homeostasis and neuroinflammation. Normally, Nrf2 exists in a dormant resting state owing to its sequestration with Keap1 in the cytoplasm (40). In response to an oxidative stimulus, Nrf2 gets separated from Keap1 and undergoes nuclear translocation, wherein it switches on the activity of anti-oxidant response element (ARE) and up-regulates Nrf2-regulated gene transcription. By doing so, it regulates the expression of various stress proteins and detoxifying enzymes, such as HO-1 and superoxide dismutase-1 (SOD-1) (41, 42). HO-1 is expressed in many neuronal cells (HT22 and BV2 cells) (43) and is known to inhibit the generation of the proinflammatory factor nitric oxide and inflammatory cytokines (29). Its ability to oppose oxidation, inflammation, and apoptosis, as well as its immunomodulatory properties, has been well documented (44). Phytochemicals and HO-1 inducers can exert protective functions against oxidative stress and inflammation through Nrf2/HO-1 modulation (45–47). Activation of Nrf2 is known to suppress transcriptional up-regulation of IL-6 and IL-1β proinflammatory cytokines in myeloid cells, mediated by binding interaction with proinflammatory genes. This phenomenon shifts the polarization of macrophages to M2-like phenotype (48, 49). Nrf2-knockout microglia exhibit up-regulated NF-κB p65 and cluster of differentiation 86 (CD86) expression, which is suggestive of an inflammatory phenotype. We demonstrate the ability of TX to activate the HO-1/Nrf2 anti-oxidant pathway, and show that it markedly induced HO-1 and gradually enhanced Nrf2 nuclear translocation (Figure 6). This phenomenon seems to indirectly regulate the neuroinflammatory response via IL-6 and TNF-α inhibition. Notably, erinacine C and amentoflavone have been shown to activate Nrf2/HO-1 signaling in BV2 cells, analogous to TX, further supporting that Nrf2-mediated HO-1 induction is a critical node for suppressing neuroinflammation (37, 38). Additionally, 4-methoxycinnamyl p-coumarate enhances Nrf2/HO-1 signaling while inhibiting NF-κB, mirroring the dual mechanism observed in TX (29).

Nrf2 activation enhances HO-1 expression and down-regulates NF-κB signaling, consequently leading to inhibition of inflammation owing to the suppression of inflammatory cytokine production, such as TNF-α (50). Nrf2 and NF-κB signaling pathways are thought to work in cooperation to maintain the physiological homeostasis and regulate cellular coping mechanisms to stress and inflammation. In comparison with wild-type control mice, Nrf2-deficient mice exhibit increased concentrations of NF-κB-regulated TNF-α, IL-1β, IL-6, and matrix metalloproteinase 9 (MMP9) pro-inflammatory molecules in response to neuroinflammation. Thus, the loss of Nrf2 expression can promote inflammation in an NF-κB-dependent manner (51). Studies thus far have underscored the vital role of neuroinflammation in the pathogenesis of AD and PD, and that the imbalance between the expression of Nrf2 and NF-κB contributes to neuroinflammation. It is also known that p65 down-regulates the transcription of Nrf2-ARE by competitively interacting with CREB-binding protein (CBP)’s cysteine/histidine-rich domain 1 (CH1) and KIX domain (CH1-KIX) and consequently inactivating Nrf2. Alternatively, it may mediate this effect via recruitment of histone deacetylase 3, a corepressor, to ARE and promotion of local histone hypoacetylation (52). Accordingly, p65 expression knockdown leads to the formation of a complex between Nrf2 and CBP. These results indicate that the TX-mediated inhibition of neuroinflammation can be modulated by blocking the activity of NF-κB and activating KEAP1-NRF2/HO-1 pathways. 

The limitation of our study is that these results can only provide partial evidence for the involvement of Nrf2 in the anti-neuroinflammatory effect of TX. However, it can not conclusively demonstrate that TX achieves anti-neuroinflammation by activating the Nrf2 signaling pathway due to the lack of targeted experiments. Further verification of the role of the Nrf2 signaling pathway in the anti-neuroinflammatory effect of TX requires *in vivo* and *in vitro* experiments using Nrf2 agonists or blockers.

**Table 1:** Primer sequences of mouse genes for RT-qPCR

Primer	Sequence
TGF-β（F）	5′-CCGCACTGTCATTCACCACCGTGTG-3′
TGF-β（R）	5′-TGCCTCGCCAAACTTCTCCAAACCG-3′
CD206（F）	5′-AGGGACGTTTCGGTGGACTGTGGAC-3′
CD206（R）	5′-TTGTGGGCTCTGGTGGGCGAGTC-3′
IL-1β（F）	5′-CCCGTGGACCTTCCAGGATGAGGAC-3′
IL-1β（R）	5′-GCAGGGTGGGTGTGCCGTCTTTC-3′
IL-6（F）	5′-CCACTTCACAAGTCGGAGGC-3′
IL-6（R）	5′-GCCACTCCTTCTGTGACTCCA-3′
β-actin（F）	5′-GCTCCGGCATGTGCAAAG-3′
β-actin（R）	5′-TTCCCACCATCACACCCTGG-3′

**Figure 1:**
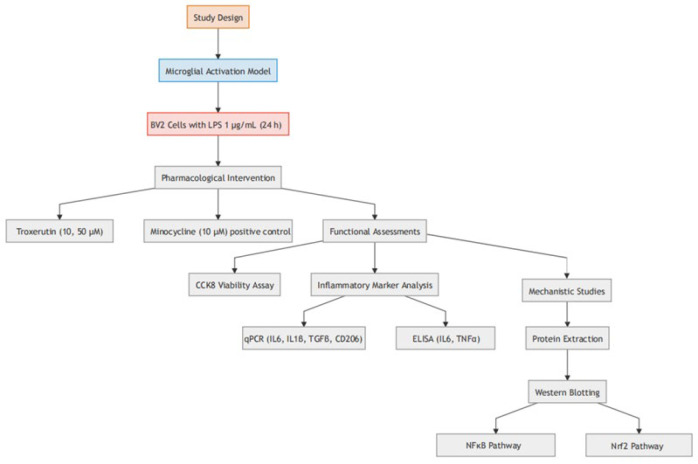
Technical roadmap of the experimental design investigating troxerutin’s anti-neuroinflammatory effects on LPS-stimulated BV2 microglia

**Figure 2:**
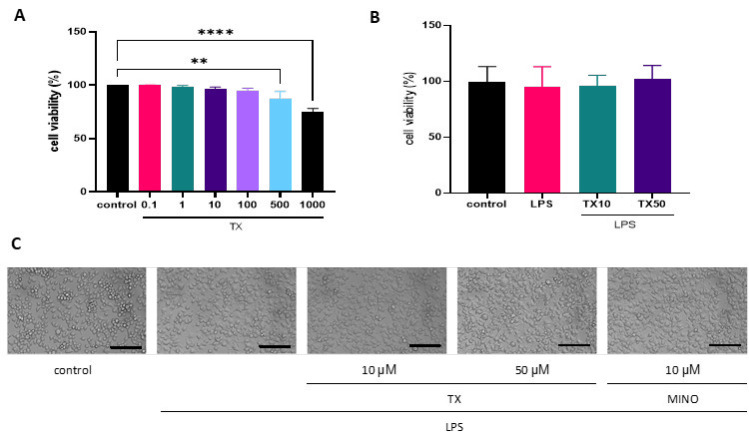
(A) Effect of TX on the viability of BV2 microglia. The data are presented as the mean ± SD of three independent experiments, **P<0.01, ****P<0.0001 versus control group. (B) Effect of TX on the viability of LPS-stimulated BV2 microglia

The data are presented as the mean ± SD of three independent experiments. (C) Impact of TX on LPS-induced morphological changes in BV2 cells

Bright-field micrographs show the changes in BV2 cell morphology following LPS exposure. Scale bar, 200 μm. TX: Troxerutin

**Figure 3:**
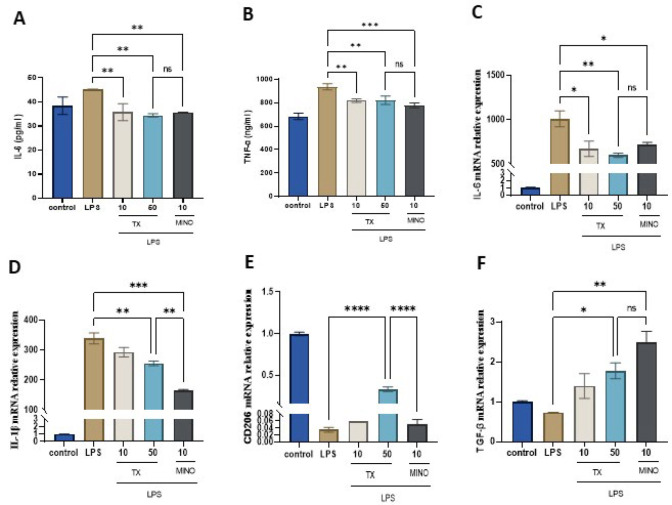
TX reduces pro-inflammatory cytokine production (A-B) and mRNA expression (C-D) in the LPS-stimulated M1 phenotypes. TX up-regulates mRNA expression of anti-inflammatory cytokines in M2 phenotypes (E-F)

The data are presented as the mean ± SD of three independent experiments, *P<0.05, **P<0.01, ***P<0.001, ****P<0.0001 versus the LPS group. TX: Troxerutin; LPS: lipopolysaccharide

**Figure 4:**
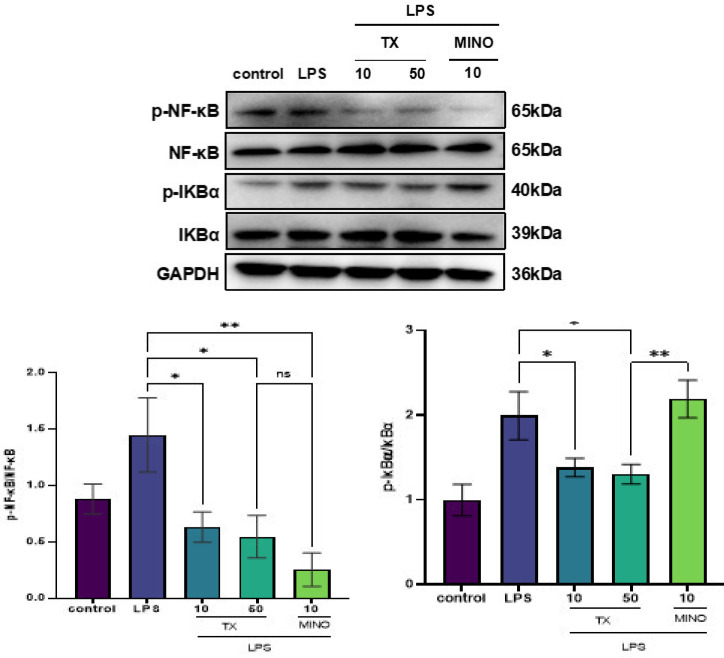
TX abrogates the NF-κB p-p65/p65 and p-IκBα/IκBα increase in BV2 cells stimulated with LPS

The data are presented as the mean ± SD of three independent experiments, *P<0.05, **P<0.01 versus LPS group. TX: Troxerutin; LPS: lipopolysaccharide

**Figure 5:**
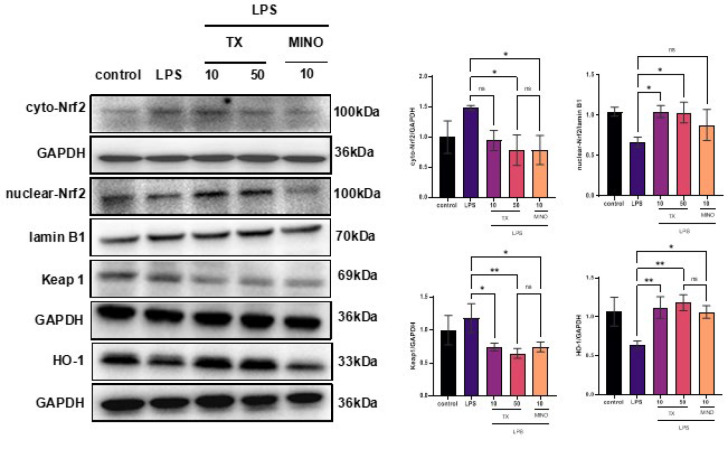
TX regulates KEAP1-NRF2/HO-1 signaling in LPS-activated BV2 microglia

The data are presented as the mean ± SD of three independent experiments, *P<0.05, **P<0.01 versus LPS group. TX: Troxerutin; LPS: lipopolysaccharide

**Figure 6:**
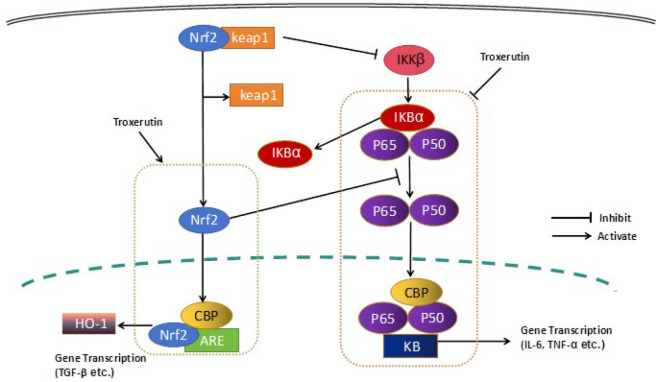
TX inhibits NF-κB signaling and accentuates HO-1/Nrf2 activation

## Conclusion

This study underscores the neurobiological properties of TX by confirming how it inhibits the LPS-induced neuroinflammatory response in BV2 microglia cells. TX abolishes the induction of pro-inflammatory molecules in BV2 microglia cells, which may be involved in the NF-κB signaling blockade and increased HO-1/Nrf2 activation. These findings emphasize the therapeutic potential of TX for inflammation-associated neurodegeneration and neurological diseases that need further exploration in animal models. 

## Declaration

We have not used any AI tools or technologies to prepare this manuscript.
